# The Chinese version of obsessive compulsive drug use scale: validation in outpatient methadone maintenance treatment program

**DOI:** 10.1186/s12888-020-02843-2

**Published:** 2020-09-25

**Authors:** Hengfen Gong, Yingying Zhang, Qihuan Ren, Zhirong Zhou, Huijing Zhou, Xirong Sun, Chencheng Zhang, Valerie Voon, Min Zhao, Shunying Yu

**Affiliations:** 1 Shanghai Mental Health Center, Shanghai Jiao Tong University School of Medicine, Shanghai, China; 2 Shanghai Pudong New Area Mental Health Center, Tongji University School of Medicine, Shanghai, China; 3 Department of Functional Neurosurgery, Ruijin Hospital, Shanghai Jiao Tong University School of Medicine, Shanghai, China; 4 Department of Clinical Psychology, Shanghai Hongkou Mental Health Center, Shanghai, China; 5 Department of Functional Clinic of the Community Based Methadone Maintenance Therapy, Shanghai Xuhui Mental Health Center, Shanghai, China; 6 Department of Drug Dependence, Shanghai Yangpu Mental Health Center, Shanghai, China; 7 Department of Psychiatry and Behavioral and Clinical Neurosciences Institute, University of Cambridge, Cambridge, UK; 8 Shanghai Key Laboratory of Psychotic Disorders, Shanghai, China; 9 Institute of Psychological and Behavioral Science, Shanghai Jiao Tong University, Shanghai, China

**Keywords:** Obsessive compulsive drug use scale, Methadone maintenance treatment, Craving, Nicotine dependence, Heroin

## Abstract

**Background:**

The Obsessive Compulsive Drug Use Scale (OCDUS) measures the overall craving level within a period from a multidimensional perspective. However, no studies have addressed the validity of the new OCDUS factor structure, presented in 2016, in China. Additionally, there is lack of evidence on the interaction among risk factors for relapse. We aimed to assess the psychometric properties of the scores of the Chinese version of the OCDUS in patients with heroin dependence receiving methadone maintenance treatment (MMT). Further, we aimed to assess the correlations of the OCDUS scores with withdrawal symptoms, depression, anxiety, and nicotine dependence.

**Methods:**

We enrolled 113 adults (age 32–64 years) and administered them with the OCDUS, Subjective Opioid Withdrawal Scale (SOWS), Beck Depression Inventory-II (BDI-II), State-Trait Anxiety Inventory (STAI), and Fagerstrom Test for Nicotine Dependence (FTND).

**Results:**

Exploratory factor analysis identified a 3-dimensional component that included “Frequency of craving,” “Inference of heroin,” and “Control of heroin.” These factors showed acceptable internal consistency, adequate item-total correlations, and significant item-subscale correlations. There was no correlation between the OCUDS scores and age, education, duration of receiving MMT, and MMT dosages. However, there was a significant correlation between the OCDUS total scores and the SOWS, STAI, BDI-II, and FTND scores. The scores of all the subscales were associated with the SOWS scores; further, the scores of the first two subscales were associated with BDI-II scores while only the scores of the first subscale were associated with the FTND scores.

**Conclusions:**

Our findings support the reliability and structure validity of the OCDUS scores. Heroin craving, withdrawal symptoms, negative emotions, and nicotine dependence, which are considered as risk factors for heroin relapse, might interact with each other. There is a need for further studies on the underlying mechanism of these clinical phenomena.

## Background

Drug addiction is a chronic relapsing disorder that is characterized by compulsions to seek and use drugs despite significant drug-related problems [1]. Craving is an important addiction feature and has been included as a diagnostic criterion for substance use disorders in the DSM-5 [2]. Generally, it is defined as a subjective experience of wanting to use a drug and is a motivational driver in addictive processes [3]. Clinical studies have used craving as an outcome measure, while mechanism studies have used it as the direct intervention, indicating its critical role in basic and clinical researches. The most common tool for assessing craving is the single-item visual analog scale (VAS), which generally assesses the craving intensity. However, it cannot assess the various craving dimensions, such as frequency or duration of craving occurrences. Therefore, a comprehensive measurement of craving is preferred for clinical and research purposes.

The Obsessive Compulsive Drug Use Scale (OCDUS) was developed by Flanken et al. (2002) to measure the overall craving level for heroin within a period from a multidimensional perspective and consisted of 13 items. It was adapted from the Obsessive Compulsive Drinking Scale (OCDS) composed of 14 items, which was modified to allow assessment of craving for other substances such as tobacco, cocaine, and cannabis [4–11]. Flanken et al. (2002) combined two items referring to the interference of consumption with working and social life from OCDS into one item in the OCDUS. The OCDUS can capture the obsessive-compulsive characteristics of drug addiction and is frequently used in western countries. Yang et al. (2016) validated the OCDUS among male heroin addicts in China and presented a different factor structure of the OCDUS from that of the original report by Flanken et al. (2002). However, there are no studies on the validity of the new OCDUS structure in China. Moreover, there are no studies on the relationships among craving, negative affect, and withdrawal, which are central in the formation and maintenance of addiction [2].

We aimed to evaluate the psychometric properties of the OCDUS in people with heroin dependence receiving methadone maintenance treatment (MMT). Further, we aimed to examine the associations of craving with withdrawal symptoms, depression, anxiety, and nicotine dependence. We hypothesized that people with a high heroin craving level have severe withdrawal symptoms, increased depression and anxiety, and a high nicotine dependence degree.

## Methods

### Participants

A total of 113 participants completed the questionnaires. Table 1 presents the participants’ characteristics. The average age and educational years of the participants were 49.9 (standard deviation [SD] = 8.15) and 10.3 (SD = 1.92), respectively, while 72.6% of the participants were men. A majority (*n* = 58, 51.3%) of the participants were married, 27.4% were divorced, and the remaining were single. Further, majority of them had a smoking habit (*n* = 110, 97.3%). Regarding the history of methadone use, the average duration of MMT was 90 months (SD = 51.4) while the mean dosage was 44.7 mg each time (SD = 28.9). Further, 82.3% of the participants had attempted to reduce their MMT dosage with 64.6% of them succeeding.

**Table 1:** Demographic characteristics of the participants

	N (%)
**Gender**	
Female	31 (27.4%)
Male	82 (72.6%)
**Marriage Status**	
Single	24 (21.3%)
Married	58 (51.3%)
Divorced	31 (27.4%)
**Smoking**	
Yes	110 (97.3%)
No	3 (2.7%)
**Attempt to reduce MMT dose**	
Yes	93(82.3%)
No	13(11.5%)
Unknown	7(6.2%)
**Successful attempt to reduce MMT dose**	
Yes	73(64.6%)
No	20(17.7%)

### Measures

#### OCDUS

The OCDUS is a self-reported scale that measures the level of craving for drugs during the past weeks. The scale used in this study was validated in Chinese heroin-dependent patients enrolled from detoxification centers [12]. It consists of 13 items with the score of each item ranging from 1 to 5; further, items 6 and 13 are reverse-scored. A higher total score indicates a higher craving level.

#### Subjective opiate withdrawal scale (SOWS)

The SOWS is a self-reported 16-item scale that assesses the presence and intensity of the opiate withdrawal experience [13]. Each item is rated on a 5-point Likert scale ranging from 0 (not at all) to 4 (extremely). A higher score indicates more severe withdrawal symptoms. The SOWS was used to measure the severity of withdrawal symptoms of the MMT patients before intake of their daily methadone dose.

#### Fagerstrom test for nicotine dependence (FTND)

The FTND is a 6-item scale for measuring nicotine dependence [14, 15]. The total score of the scale ranges from 0 to 10, with a higher score indicating stronger dependence.

#### Beck depression inventory-II (BDI-II)

The BDI-II is a self-administrated 21-item scale for assessing the presence of depression [16]. The score of each item ranges from 0(absent) to 3(severe), with a higher score indicating a more severe depression severity.

#### State-trait anxiety inventory (STAI)

The STAI consists of two 20-item self-reported subscales, namely, the STAI State (STAI-S) and STAI Trait (STAI-T) [17]. The STAI-S assesses the transient momentary emotional status, while the STAI-T assesses the general reaction in stressful situations. Each item is rated on a 4-point Likert scale that ranges from 1 (not at all) to 4 (very much so) for the STAI-S and 1 (almost never) to 4 (almost always) for the STAI-T. The score range for each subscale is 20–80, with a higher score indicating a greater degree of anxiety.

### Procedure

The study was approved by the Ruijin Hospital Ethics Committee of Shanghai Jiao Tong University School of Medicine. All procedures were performed in accordance with the ethical standards of the responsible committee on human experimentation (institutional and national) and the Helsinki Declaration of 1975, as revised in 2008. We enrolled participants from 3 MMT clinics in Shanghai. The inclusion criteria were as follows: (1) sufficient reading proficiency to understand and complete the questionnaires; (2) having received MMT for at least 1 month; (3) ability to provide written consent.

All the enrolled participants provided written informed consent. Subsequently, an experimenter instructed the participants to complete the self-reported scales in a quiet room. The experimenter checked the completed scales for blank entries for each item.

### Data analysis

We conducted a descriptive analysis to examine the demographic characteristics and the scales’ scores. We used the Kolmogorov-Smirnov one-sample test to assess whether the scales’ scores were normally distributed. We conducted exploratory principal components analysis with varimax rotation to determine the factor structure of the OCDUS. We conducted confirmatory factor analysis (CFA) to investigate the model fitness based on the exploratory factor analysis (EFA) findings. We used the maximum likelihood chi-square statistic, the comparative fit index (CFI), and the root mean square error of approximation (RMSEA) to assess the goodness-of-fit of the model. A χ^2^/df between 1.0 and 3.0 was considered acceptable. Further, an RMSEA of 0 to 0.05 and 0.05 to 0.08 indicated a good and acceptable fit, respectively. We considered a CFI of > 0.95 a good fit. We removed items with a standardized regression weight of < 0.5. Pearson’s correlation coefficients were used to examine the item-subscale and item-total correlations of the OCDUS. We computed Cronbach’s α to assess the internal consistency of the OCDUS. We analyzed the Pearson’s correlations between the OCDUS and the SOWS, BDI-II, STAI, and FTND. The association between the OCDUS score and the previous attempt to decrease methadone dosage was tested by the comparison of mean scores of the OCDUS with a T test. We conducted statistical analyses using SPSS 18.0 with statistical significance set at a two-tailed value of *p* < 0.05.

## Results

### EFA of the OCDUS

Kaiser-Meyer-Olkin (KMO) and Bartlett’s tests indicated that the data were suitable for factor analysis (KMO = 0.851, *p* < .000). We conducted principal component analysis to determine the factor structure of the 13-item OCDUS. The first three eigenvalues were 5.66, 2.23, and 1.21. This three-factor model accounted for 69.26% of the OCDUS-item variance. Table 2 presents the descriptive statistics for each OCDUS item, as well as the communalities and factor loadings for the three-factor model. All items had salient (≥ 0.40) loadings on the five factors (range from 0.59 to 0.86). The three-factor model was similar to the factor model reported by Yang et al. (2016). We measured the first factor using items 1, 2, 7, 8, 10, and 12; the second factor using items 3, 4, 5, and 9; and the third factor using items 6, 11, and 13.

**Table 2:** The Obsessive-Compulsive Drug Use Scale (OCDUS): Means (M), standard deviations (SD), factor loadings, communalities (h^2^), and Cronbach’s α for the three-factor model

			Factor loadings				Cronbach’s α(item deletion)	The Chinese OCDUS
Item	M	SD	1	2	3	h^**2**^		
8	1.74	1.14	**0.85**	0.15	0.14	0.76	0.869	1
12	1.53	0.96	**0.84**	0.11	0.08	0.73	0.875	1
2	2.01	1.00	**0.83**	0.25	0.07	0.76	0.867	1
7	1.96	1.03	**0.82**	0.24	0.06	0.74	0.869	1
10	2.29	1.31	**0.70**	0.26	−0.03	0.56	0.900	2
1	1.90	1.13	**0.67**	0.28	0.08	0.54	0.891	1
4	2.40	1.29	0.36	**0.86**	0.01	0.87	0.655	2
3	2.15	1.22	0.33	**0.86**	0.07	0.85	0.672	2
9	2.36	1.30	0.46	**0.60**	0.00	0.58	0.783	R
5	2.98	1.37	0.00	**0.59**	−0.56	0.66	0.866	2
13	2.58	1.39	0.12	0.12	**0.85**	0.75	0.391	3
11	2.64	1.45	0.12	0.27	**−0.75**	0.66	0.669	2
6	3.46	1.40	0.29	0.22	**0.65**	0.56	0.618	3

R: removed by Yang et al. 2016. The last column indicates the allocation of items among factors based on Yang et al. 2016

### CFA of the OCDUS

The CFA results indicated that the fit indices of the EFA-derived three-factor model were unacceptable for the OCDUS (χ^2^ = 167.36, df = 62, *p* < .000, RMSEA = 0.123, CFI = 0.872). The standardized regression weights of items 5 and 11 were all < 0.5; specifically, 0.427 and − 0.482, respectively. The standardized regression weights of the other items ranged from 0.579 to 0.936. We deleted items 5 and 11 due to their unacceptable standardized regression weights. Further, we deleted item 10 since it evaluated aspects different from those of other items of the first factor. Therefore, the final first factor consisted of items 1, 2, 7, 8, and 12; the second factor consisted of items 3, 4, and 9; and the third factor included items 6 and 13. Moreover, the CFA results supported the final three-factor model (χ^2^ = 39.29, df = 32, *p* < .000, RMSEA = 0.045, CFI = 0.989). The factor loadings on the three factors ranged from 0.54 to 0.93 (Table 3). Based on the study by Yang et al., the three factors were named as “Frequency of Craving (FR),” Interference of Heroin (IH),” and “Control of Heroin (CH).” In the further analysis, items 5, 10, and 11 were not included.

**Table 3:** The standardized factor loadings of each item on the corresponding factor and the item-total and item-subscale correlation of the OCDUS

Item	Loading	Item-totalcorrelation	Item-subscalecorrelation
**Frequency of craving (FR)**			
1	0.71	0.70	0.79
2	0.90	0.80	0.90
7	0.81	0.78	0.84
8	0.83	0.78	0.87
12	0.80	0.74	0.85
**Inference of heroin (IH)**			
3	0.91	0.74	0.92
4	0.93	0.75	0.92
9	0.66	0.68	0.83
**Control of heroin (CH)**			
6	0.93	0.57	0.87
13	0.54	0.44	0.86

### Reliability of the OCDUS

The internal consistency of the OCDUS was acceptable (α = 0.87) with Cronbach’s α of 0.90, 0.87, and 0.67 for FR, IH, CH, respectively. The Kolmogorov-Smirnov test revealed no significant difference of the distribution of the 10-item OCDUS scores from a normal distribution (z [113] = 1.21, *p* = 0.11). The 10-item OCDUS showed adequate item-total correlations (mean [M] = 0.70, range = 0.44–0.80, Table 3), and significant item-subscale correlations (M = 0.87, range = 0.84–0.92). There were significant correlations of the 10-item OCDUS score with the subscales scores; further, there were significant correlations between the subscale scores (Table 4).

**Table 4:** The descriptive statistics of the OCDUS and other measures and the Pearson correlations between them

	The OCDUS total score	FR	IH	CH	M	SD	Range
The OCDUS	1				21.3	8.21	10–42
FR Subscale	0.90***	1			9.2	4.46	5–23
IH Subscale	0.82***	0.61***	1		6.9	3.39	3–15
CH Subscale	0.59***	0.35***	0.24*	1	5.2	2.46	2–10
Age	0.05	−0.01	0.01	0.16	49.9	8.15	32–64
Education Years	−0.13	−0.17	−0.15	0.07	10.3	1.92	6–16
Duration of receiving MMT (months)	−0.02	−0.01	− 0.02	−0.02	90.1	51.39	2–224
Current Methadone Dosage (ml)	−0.01	−0.01	− 0.11	0.15	44.7	28.92	2–150
SOWS	0.61***	0.58***	0.49***	0.31***	10	11.25	0–50
FTND	0.31***	0.35***	0.18	0.17	4.9	2.55	0–10
BDI-II	0.44***	0.41***	0.47***	0.08	14.1	10.95	0–18
STAI-S	0.52***	0.49***	0.40***	0.30**	36.7	11.34	20–68
STAI-T	0.49***	0.42***	0.48***	0.21*	42.2	10.96	21–71

^*^p < 0.05, ^**^p < 0.01, ^***^p < 0.001

### Correlations among the OCDUS, demographic variables, and other measures

Table 4 presents the descriptive statistics and the correlations among the OCDUS, demographic variables, and other measures. There was no significant correlation of the OCDUS scores with age (r = − 0.05, *p* = 0.63), educational years (r = − 0.13, *p* = 0.17), duration of receiving MMT (r = − 0.02, *p* = 0.83), and current methadone dosages (r = − 0.01, *p* = 0.93). There was no difference in the OCDUS total scores between patients who wanted to reduce their methadone dosage and those who did not want to reduce their methadone dosage (t = − 1.69, *p* = 0.09). There was a significant correlation of the SOWS scores with the OCDUS total scores and the three subscales scores (r = 0.31–0.61, *p* < 0.001). The FTND scores were significantly correlated with the OCDUS and FR scores (r = 0.31, 0.35, *p* < 0.001) but not with the IH (r = 0.18, *p* = 0.05) and CH (r = 0.17, *p* = 0.07) scores. There was a significant correlation of the BDI-II, STAI-S, and STAI-T scores with the OCDUS, FR, and IH scores (r = 0.40–0.52, *p* < 0.001). The CH scores were not significantly correlated with the BDI-II scores (r = 0.08, *p* = 0.42); however, they were moderately correlated with the STAI-S (r = 0.30, *p* = 0.001) and STAI-T (r = 0.21, *p* = 0.03) scores.

## Discussion

We aimed to examine the psychometric properties of the OCDUS and its associations with withdrawal, emotions, and nicotine dependence. We found that the scale had good structure validity with the deletion of items 5, 10, and 11; finally, we included 10 items with three subscales, namely, FR, IH, and CH. The 10-item scale showed acceptable internal consistency, adequate item-total correlations, and significant item-subscale correlations. There were significant positive correlations of the OCDUS total scores with the SOWS, BDI, STAI-S, STAI-T, and FTND scores. Moreover, there was an obvious correlation of the SOWS, STAI-S, and STAI-T scores with the scores of the three subscales. Furthermore, the BDI-II scores were correlated with the scores of the FR and IH subscales; however, the FTND scores were only correlated with the FR subscale scores.

Previous findings on the OCDUS components were inconsistent. Flanker et al. (2002) conducted an exploratory factor analysis of 102 inpatients and obtained three factors, namely, “heroin thoughts and interference,” “desire and control,” and “resistance to thoughts and intention.” Finally, they obtained a 12-item scale with deletion of item 10 and modified it to measure cocaine craving in Dutch individuals and acquired a similar factor structure in 101 cocaine-dependent inpatients [8]. Vorspan et al. (2012) tested 116 French-speaking cocaine users and revealed a three-factor structure scale, which was similar to the three factors of the original OCDS (Roberts et al. 1999). However, another study conducted in the United States supported the two-factor structure; it comprised of “obsessive” and “compulsive” in 107 cocaine-dependent participants [6]. A French translation of the OCDS also developed a two-factor structure in 74 native French-speaking alcohol-dependent patients [18]. Moreover, the sample size was larger in studies identifying the three-factor structure. Further, 131 male crystalline heroin abusers were administered with the Persian version of the OCDUS consisting of four components namely, “desire and mental preoccupation with drugs,” “the effect of desire for drug and drug-related thoughts on the patient’s work and life,” “motivation, emotion, and lack of control,” and “resistance to drug use” [19]. Moreover, the factor structure of the Chinese version of the OCDUS, which was validated by Yang et al. (2012) and differed from that of versions of other countries, was consistent with our study’s components. Therefore, the OCDUS components are consistent within similar countries but differ across different countries, which might be explained by linguistic and cultural differences.

Our study revealed that the craving level was positively associated with withdrawal symptoms and negative emotions in patients with heroin dependence receiving MMT. These associations could indicate good criterion validity, which is suggested by the fact that this scale assesses different craving aspects [20]. However, Yen et al. evaluated the Desire for Drug Questionnaire in patients with MMT, which is a frequently used instrument for measuring the level of instant craving for heroin, and reported that craving was not associated with withdrawal symptoms; rather, it was related to depression [21]. This inconsistency might have resulted from the different time periods for measuring the craving level. This study assessed the general craving during a certain time period; further, the scales were administered prior to daily methadone intake. Moreover, craving, withdrawal symptoms, anxiety, and depression might increase the risk for relapse of heroin use. There is a need for further studies on the underlying mechanism of these associations to promote a better understanding of the addictive process, maintenance, and relapse.

Notably, 97.3% of our participants were smokers, and the craving frequency was positively associated with nicotine dependence. Previous studies have also reported a high percentage of smokers among heroin users [12, 22, 23]. Moreover, there was an association of nicotine dependence with withdrawal symptoms and anxiety. The high smoking prevalence among patients receiving MMT might be explained by the interactive effects of addictive substances such as methadone, heroin, and nicotine [24]. However, the sample size in this study is not large enough for a reliable inference. Therefore, the validity of the OCDUS and the associations between craving and nicotine dependence should be further explored in a larger sample.

Our study has several limitations. First, we did not use a VAS as another self-reporting tool for measuring drug craving. Moreover, we did not determine the test-retest reliability. Third, we did not follow up on the participants to observe the relapse frequency. Future clinical studies should further explore the craving impacts in large representative samples.

## Conclusion

The Chinese version of the OCDUS demonstrated good reliability and structural validity and can be used in future studies. Heroin craving is associated with withdrawal symptoms, depression, anxiety, and nicotine dependence. Further studies are needed to explore the underlying mechanisms of these clinical phenomena.

## Data Availability

The data that support the findings of this study are available from the corresponding author upon reasonable request.

## Abbreviations

OCDUS: Obsessive compulsive drug use scale
MMT: Methadone maintenance treatment
SOWS: Subjective opioid withdrawal scale
BDI-II: Beck depression inventory-II
STAI: State-trait anxiety inventory
FTND: Fagerstrom test for nicotine dependence
VAS: Visual analog scale
CFA: Confirmatory factor analysis
EFA: Exploratory factor analysis
RMSEA: Root mean square error of approximation
CFI: Comparative fit index
KMO: Kaiser-Meyer-Olkin
FR: Frequency of craving
IH: Interference of heroin
CH: Control of heroin

## References

[1] Koob GF, Volkow ND (2016). Neurobiology of addiction: a neurocircuitry analysis. Lancet Psychiatry.

[2] Sayette MA (2016). The role of craving in substance use disorders: theoretical and methodological issues. Annu Rev Clin Psychol.

[3] Kakko J, Alho H, Baldacchino A, Molina R, Nava FA, Shaya G (2019). Craving in opioid use disorder: from neurobiology to clinical practice. Front Psychiatry.

[4] Anton RF, Moak DH, Latham P (1995). The obsessive compulsive drinking scale: a self-rated instrument for the quantification of thoughts about alcohol and drinking behavior. Alcohol Clin Exp Res.

[5] Roberts JS, Anton RF, Latham PK, Moak DH (1999). Factor structure and predictive validity of the obsessive compulsive drinking scale. Alcohol Clin Exp Res.

[6] Hormes JM, Coffey SF, Drobes DJ, Saladin ME (2012). The obsessive compulsive cocaine use scale: development and initial validation of a self-rated instrument for the quantification of thoughts about cocaine use. Drug Alcohol Depend.

[7] Franken IHA, Hendriks VM, Van den Brink W (2002). Initial validation of two opiate craving questionnaires: the obsessive compulsive drug use scale and the desires for drug questionnaire. Addict Behav.

[8] Lievaart M, Erciyes F, Van Der Veen FM, Van De Wetering BJM, Muris P, Franken IHA (2015). Validation of the cocaine versions of the obsessive compulsive drug use scale and the desires for drug questionnaire. Am J Drug Alcohol Abuse..

[9] Hitsman B, Shen BJ, Cohen RA, Morissette SB, Drobes DJ, Spring B (2010). Measuring smoking-related preoccupation and compulsive drive: evaluation of the obsessive compulsive smoking scale. Psychopharmacology.

[10] Dekker N, Koeter M, Van Den Brink W (2012). Craving for cannabis in patients with psychotic disorder, their non-affected siblings and healthy controls: psychometric analysis of the obsessive compulsive drug use scale. Int J Methods Psychiatr Res.

[11] Vorspan F, Bellais L, Romo L, Bloch V, Neira R, Lépine JP (2012). The obsessive-compulsive cocaine scale (OCCS): a pilot study of a new questionnaire for assessing cocaine craving. Am J Addict.

[12] Yang C, Wei W, Vrana KE, Xiao Y, Peng Y, Chen D (2016). Validation of the obsessive compulsive drug use scale (OCDUS) among male heroin addicts in China. Int J Ment Health Addict.

[13] Handelsman L, Cochrane KJ, Aronson MJ, Ness R, Rubinstein KJ, Kanof PD (1987). Two new rating scales for opiate withdrawal. Am J Drug Alcohol Abuse.

[14] Huang CL, Lin HH, Wang HH (2006). The psychometric properties of the Chinese version of the Fagerstrom test for nicotine dependence. Addict Behav.

[15] Fagerström KO (1978). Measuring degree of physical dependence to tobacco smoking with reference to individualization of treatment. Addict Behav.

[16] Dozois DJA, Dobson K, Ahnberg JL (1998). A psychometric evaluation of the Beck depression inventory–II. Psychol Assess.

[17] Julian LJ (2011). Measures of anxiety: state-trait anxiety inventory (STAI), Beck anxiety inventory (BAI), and hospital anxiety and depression scale-anxiety (HADS-A). Arthritis Care Res.

[18] Ansseau M, Besson J, Lejoyeux M, Pinto E, Landry U, Cornes M (2000). A French translation of the obsessive-compulsive drinking scale for craving in alcohol-dependent patients: a validation study in Belgium, France, and Switzerland. Eur Addict Res.

[19] Hassani-Abharian P, Mokri A, Ganjgahi H, Oghabian M-A, Ekhtiari H (2016). Validation for Persian versions of “desire for drug questionnaire” and “obsessive compulsive drug use scale” in heroin dependents. Arch Iran Med.

[20] Rosenberg H (2009). Clinical and laboratory assessment of the subjective experience of drug craving. Clin Psychol Rev.

[21] Yen CF, Lin HC, Wang PW, Ko CH, Lee KH, Hsu CY (2016). Heroin craving and its correlations with clinical outcome indicators in people with heroin dependence receiving methadone maintenance treatment. Compr Psychiatry.

[22] Pajusco B, Chiamulera C, Quaglio G, Moro L, Casari R, Amen G (2012). Tobacco addiction and smoking status in heroin addicts under methadone vs. buprenorphine therapy. Int J Environ Res Public Health.

[23] Do HP, Nguyen LH, Thi Nguyen NP, Ngo C, Thi Nguyen HL, Le GT (2017). Factors associated with nicotine dependence during methadone maintenance treatment: findings from a multisite survey in Vietnam. BMJ Open.

[24] Talka R, Tuominen RK, Salminen O (2015). Methadone’s effect on nAChRs - a link between methadone use and smoking?. Biochem Pharmacol.

